# Supplementary material for: Stromal fibroblast growth factor 2 reduces the efficacy of bromodomain inhibitors in uveal melanoma
Source: EMBO Mol Med. 2019 Jan 4;11(2):e9081. doi: 10.15252/emmm.201809081 (PMC6365926; doi:10.15252/emmm.201809081)
Supplement: Supplementary file 1 — Appendix [file EMMM-11-e9081-s001.pdf]

Table of Contents

	Page(s)
Appendix Figures S1 – S8	2-10
Appendix Figure Legends	11-12

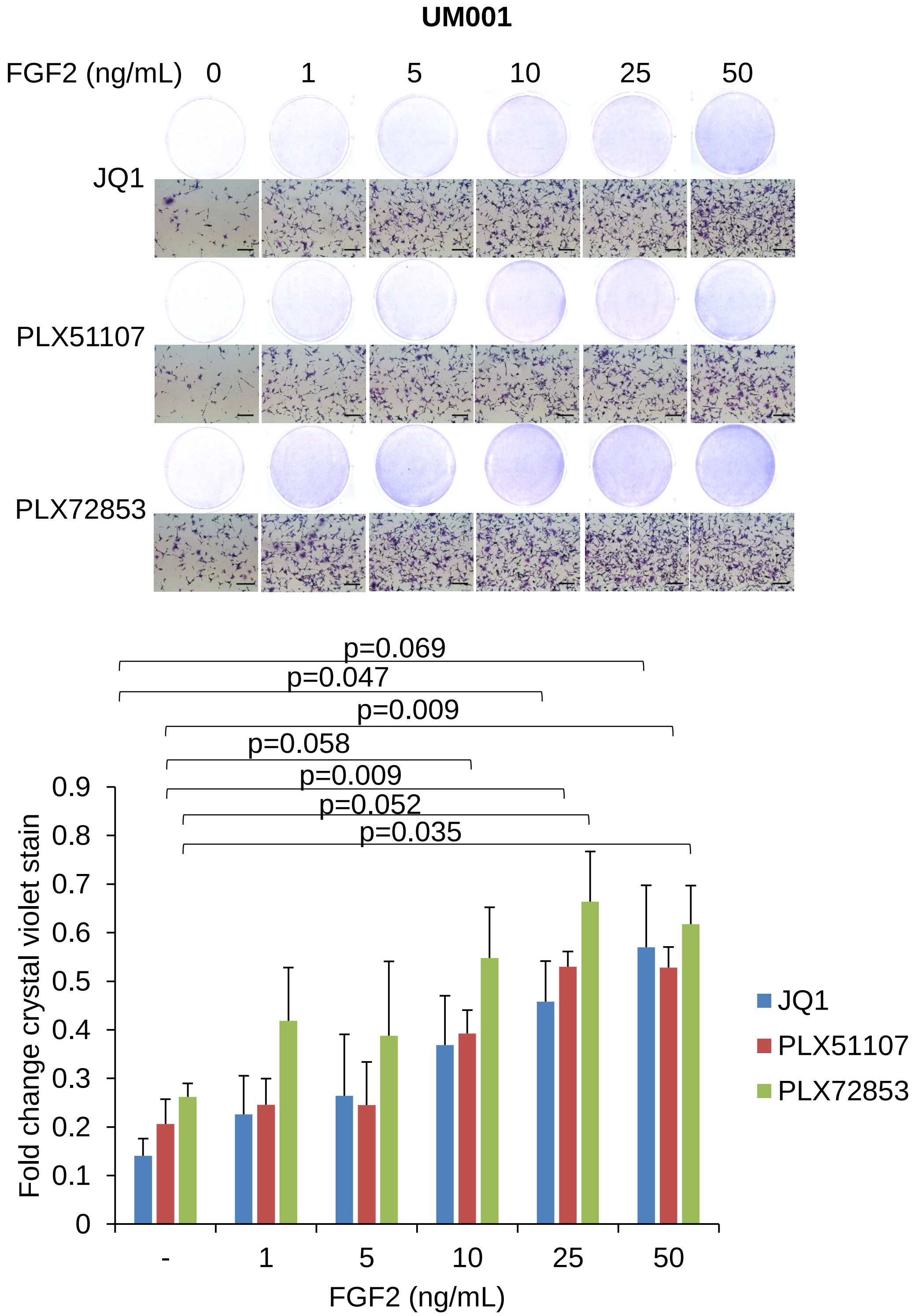
UM001
FGF2 (ng/mL) 0 1 5 10 25 50
JQ1
PLX51107
PLX72853
p=0.069
p=0.047
p=0.009
p=0.058
p=0.009
p=0.052
p=0.035
Fold change crystal violet stain
0.9
0.8
0.7
0.6
0.5
0.4
0.3
0.2
0.1
0
-
1
5
10
25
50
FGF2 (ng/mL)
JQ1
PLX51107
PLX72853

Appendix Figure S2

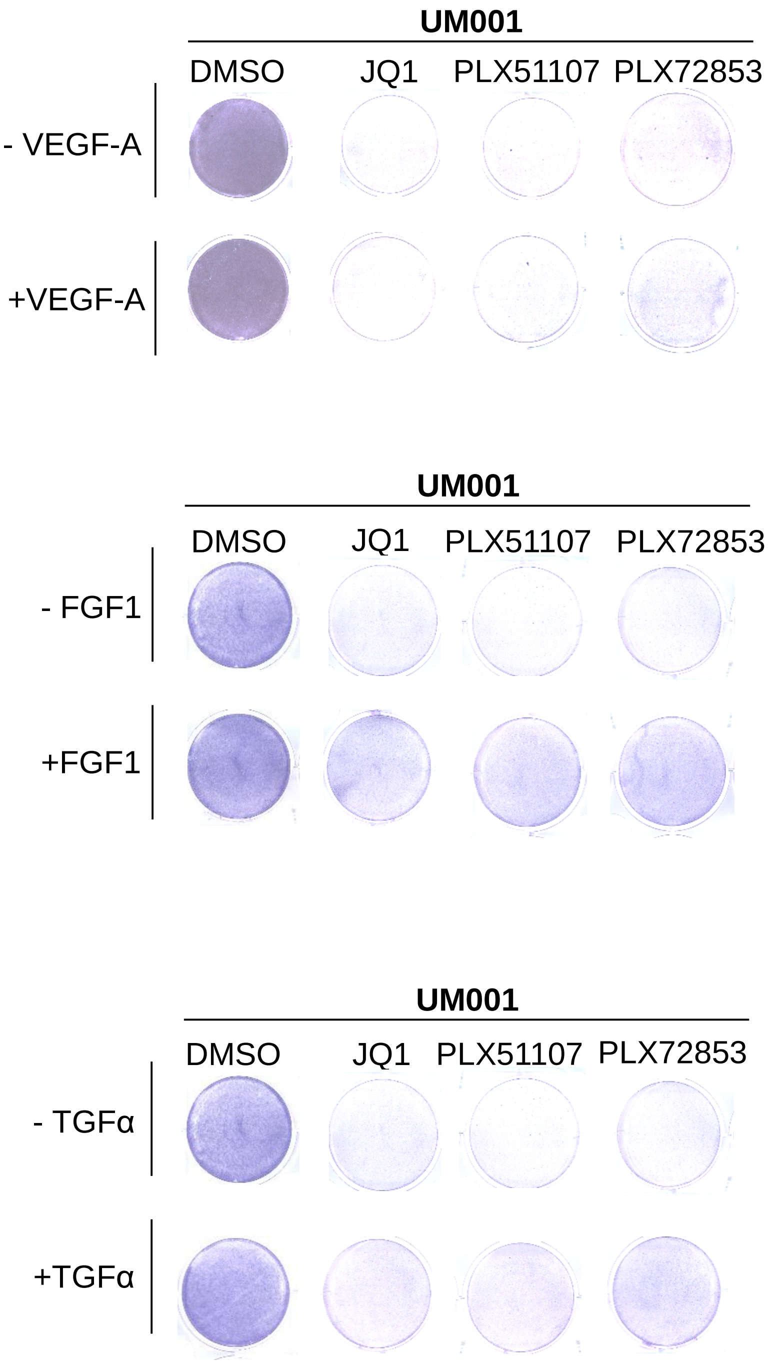

Appendix Figure S3

UM001

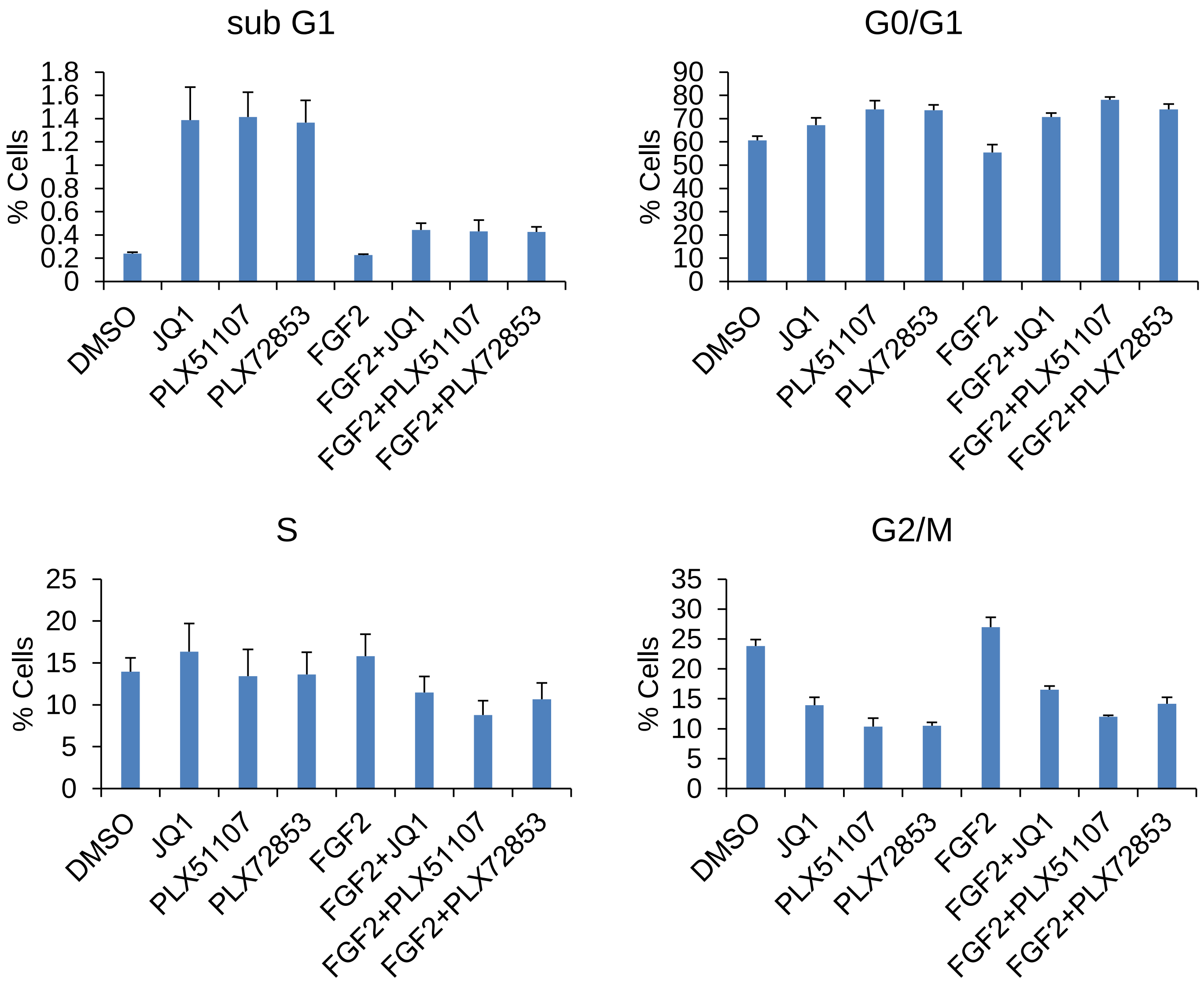

Appendix Figure S4

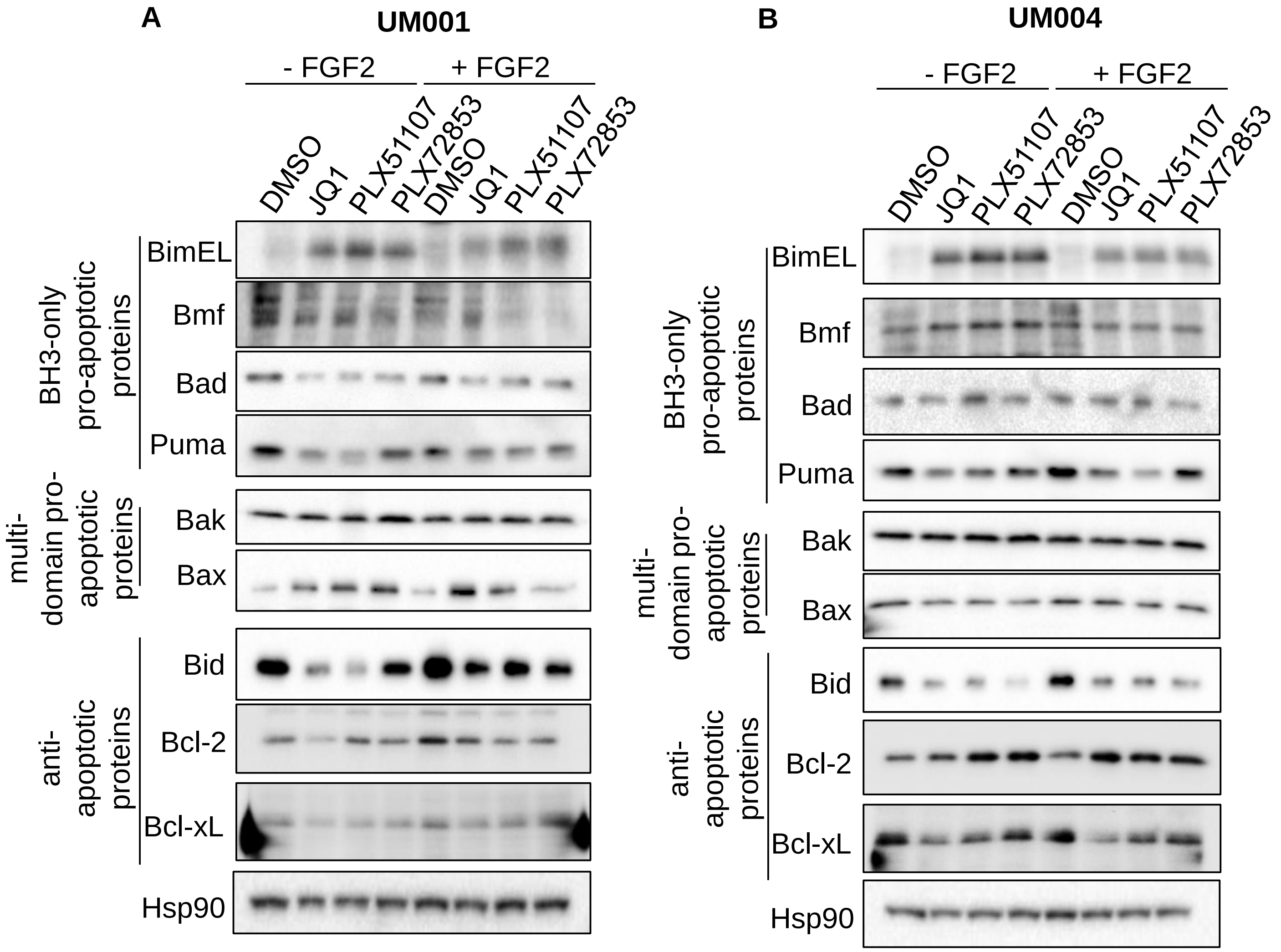

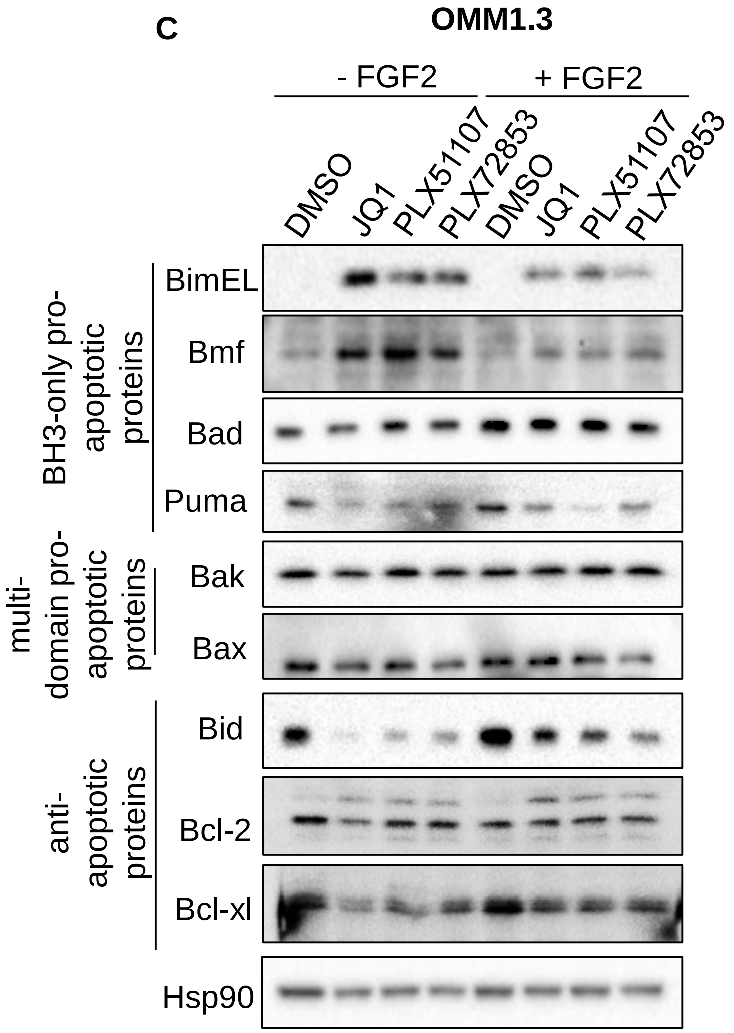
C
OMM1.3
- FGF2
+ FGF2
DMSO
JQ1
PLX51107
PLX72853
DMSO
JQ1
PLX51107
PLX72853
BH3-only pro-apoptotic proteins
BimEL
Bmf
Bad
Puma
multi-domain pro-apoptotic proteins
Bak
Bax
anti-apoptotic proteins
Bid
Bcl-2
Bcl-xl
Hsp90

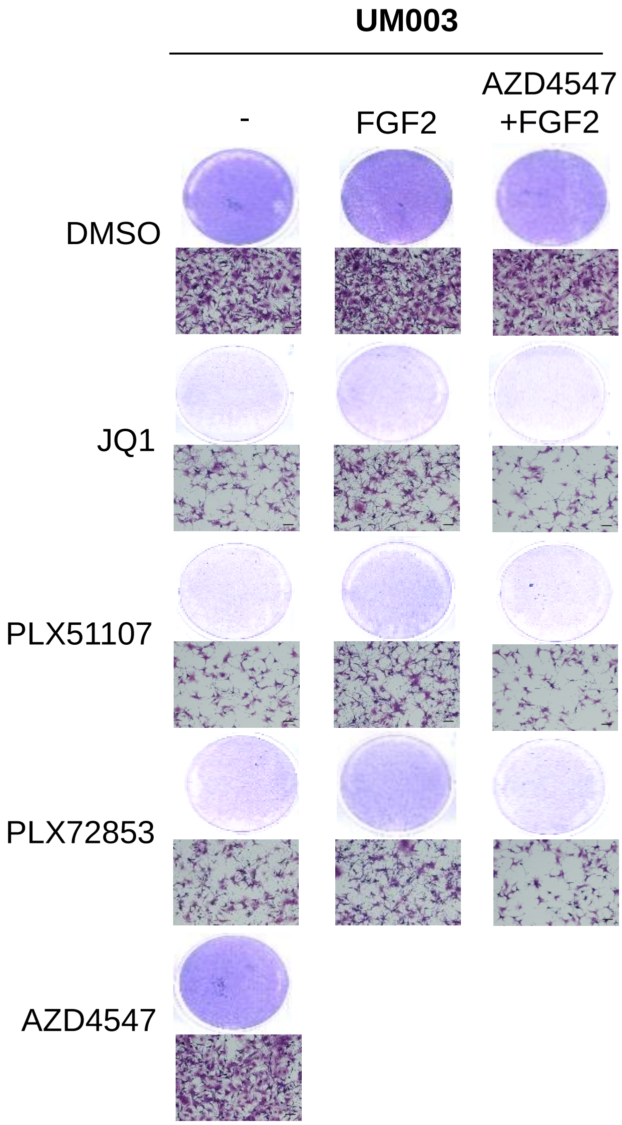
UM003
-
FGF2
AZD4547
+FGF2
DMSO
JQ1
PLX51107
PLX72853
AZD4547

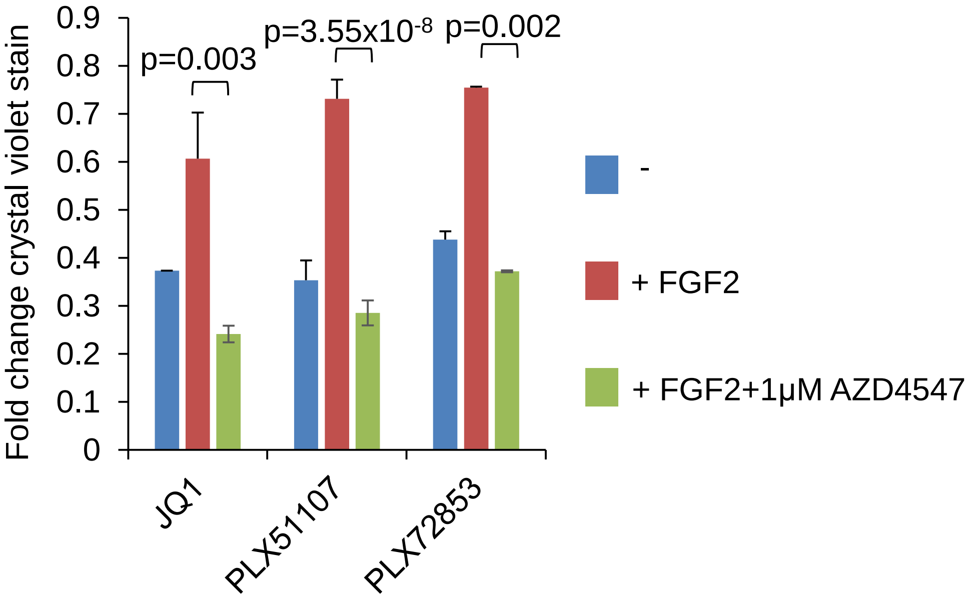
Fold change crystal violet stain
0.9
0.8
0.7
0.6
0.5
0.4
0.3
0.2
0.1
0
p=0.003
p=3.55x10-8
p=0.002
JQ1
PLX51107
PLX72853
-
+ FGF2
+ FGF2+1µM AZD4547

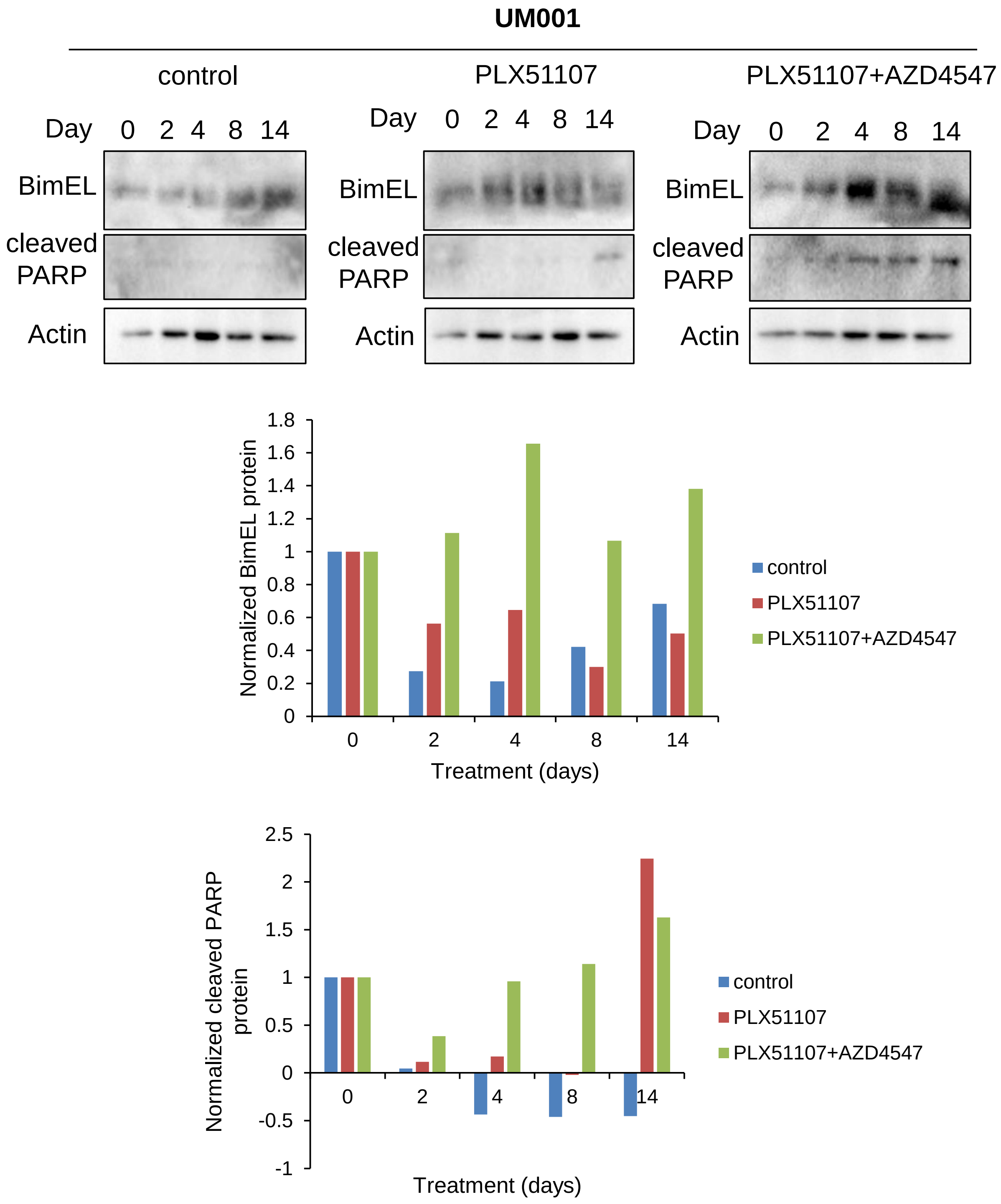
UM001
control
PLX51107
PLX51107+AZD4547
Day 0 2 4 8 14
BimEL
cleaved PARP
Actin
Normalized BimEL protein
Treatment (days)
control
PLX51107
PLX51107+AZD4547
Normalized cleaved PARP protein
Treatment (days)
control
PLX51107
PLX51107+AZD4547

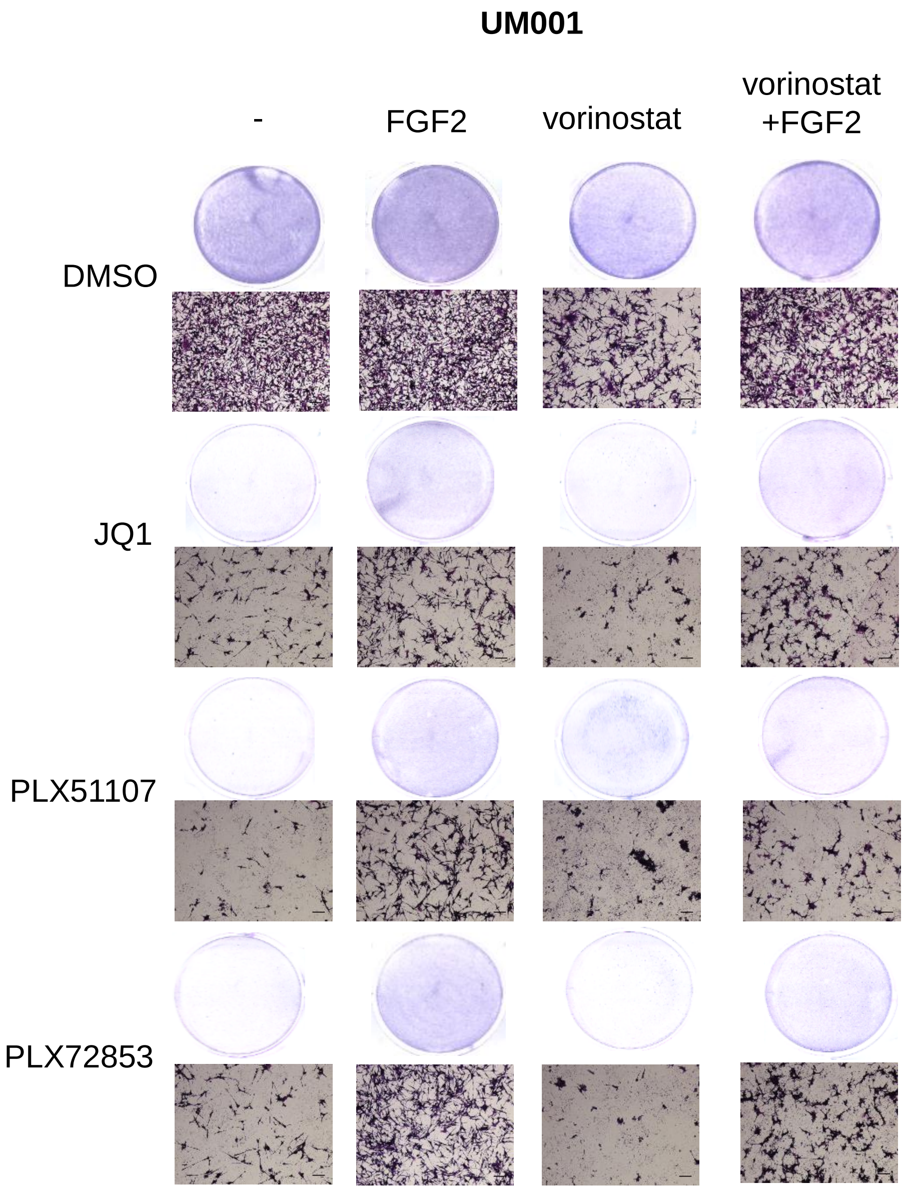
UM001
-
FGF2
vorinostat
vorinostat +FGF2
DMSO
JQ1
PLX51107
PLX72853

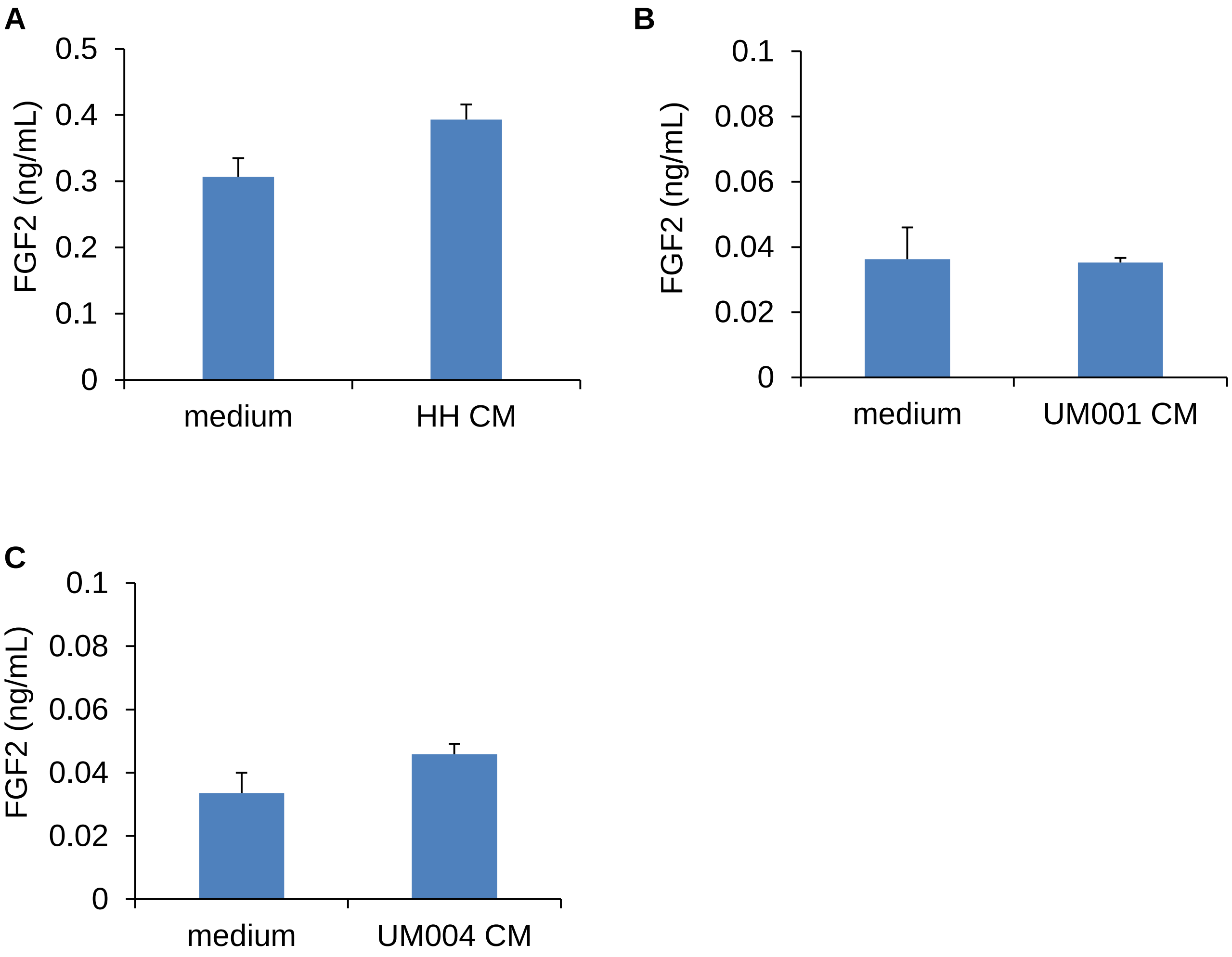
A
FGF2 (ng/mL)
0.5
0.4
0.3
0.2
0.1
0
medium
HH CM
B
FGF2 (ng/mL)
0.1
0.08
0.06
0.04
0.02
0
medium
UM001 CM
C
FGF2 (ng/mL)
0.1
0.08
0.06
0.04
0.02
0
medium
UM004 CM

Appendix Figure Legends

Appendix Figure S1: UM001 was treated with 1μM JQ1, 1μM PLX51107 or 100nM PLX72853 in combination with increasing concentrations of FGF2. Colony growth was monitored after 8 days of treatment with crystal violet. Fold change in crystal violet stain compared to DMSO and mean ± S.E.M. of data from triplicate experiments (or n=3) is shown. Statistical significance is measured using the unpaired t-test. Scale bar: 100μm.

Appendix Figure S2: Effects of VEGF-A, FGF1 and TGFα on the responses of UM001 to BET inhibitors. UM001 was treated with 1μM JQ1, 1μM PLX51107 or 100nM PLX72853 and 50ng/mL of VEGF-A, FGF1 or TGFα for 8 days. Colony growth assays using crystal violet were performed and representative images are shown.

Appendix Figure S3: PI labeling of UM001 following BET inhibitor and FGF2 treatment for 48 hours. UM001 was treated with 1μM JQ1, 1μM PLX51107 or 100nM PLX72853 and 50ng/mL FGF2. Percentage cells in sub G1, G0/G1, S and G2/M phases were detected by flow cytometry after staining UM001 with PI for 30 minutes. The mean of percentage cells from n=3 experiments is shown.

Appendix Figure S4: Characterization of apoptotic and anti-apoptotic marker expression by western blotting following treatment of (A) UM001, (B) UM004 and (C) OMM1.3 with BET inhibitors and 50ng/mL FGF2 for 48 hours. UM001 and OMM1.3 cells were treated with 1μM JQ1, 1μM PLX51107 or 100nM PLX72853, and UM004 was treated with 2μM JQ1, 2μM PLX51107 or 200nM PLX72853. Blots shown are representatives from three replicate experiments.

Appendix Figure S5: UM003 was treated with 1μM JQ1, 1μM PLX51107 or 100nM PLX72853, 50ng/mL FGF2 and 1μM AZD4547 for 8 days. Colony growth was detected by crystal violet

staining. Fold change in crystal violet stain compared to DMSO and mean ± S.E.M. of data from triplicate experiments (or n=3) is shown. The unpaired t-test was used for statistical significance. Scale bar: 100μm.

Appendix Figure S6: BimEL and cleaved PARP protein expression over 14 days of treatment of mice bearing UM001 xenografts with 90mg/kg PLX51107 and 5mg/kg AZD4547. Tumor xenografts were from subcutaneous injection of UM001 in nude mice. Representative blots are shown. BimEL and cleaved PARP protein levels were normalized to actin.

Appendix Figure S7: UM001 was treated with 1μM JQ1, 1μM PLX51107 or 100nM PLX72853, 50ng/mL FGF2 and 1μM vorinostat. Changes in colony growth were detected after 8 days of treatment by crystal violet staining. Scale bar: 100μm.

Appendix Figure S8: FGF2 levels in medium conditioned by (A) human hepatocytes (HH), (B) UM001 and (C) UM004. Conditioned medium was collected three times and FGF2 concentration was measured by ELISA. Data shown is mean ± S.E.M. of results from triplicates of a representative experiment.
