# Supplementary material for: Stromal fibroblast growth factor 2 reduces the efficacy of bromodomain inhibitors in uveal melanoma
Source: EMBO Mol Med. 2019 Jan 4;11(2):e9081. doi: 10.15252/emmm.201809081 (PMC6365926; doi:10.15252/emmm.201809081)
Supplement: Supplementary file 2 — Expanded View Figures PDF [file EMMM-11-e9081-s002.pdf]

Expanded View Figures

Figure EV1. RPPA analysis of BET inhibitor- and FGF2-induced changes in protein expression.

A, B RPPA heat map of protein expression in UM001, UM004 and OMM1.3 cells treated with (A) BET inhibitors alone or (B) in combination with 50 ng/ml FGF2 for 48 h. UM001 and OMM1.3 cells were treated with 1 μM JQ1, 1 μM PLX51107 or 100 nM PLX72853, and UM004 cells were treated with 2 μM JQ1, 2 μM PLX51107 or 200 nM PLX72853. Each condition is in triplicate.

© 2019 The Authors

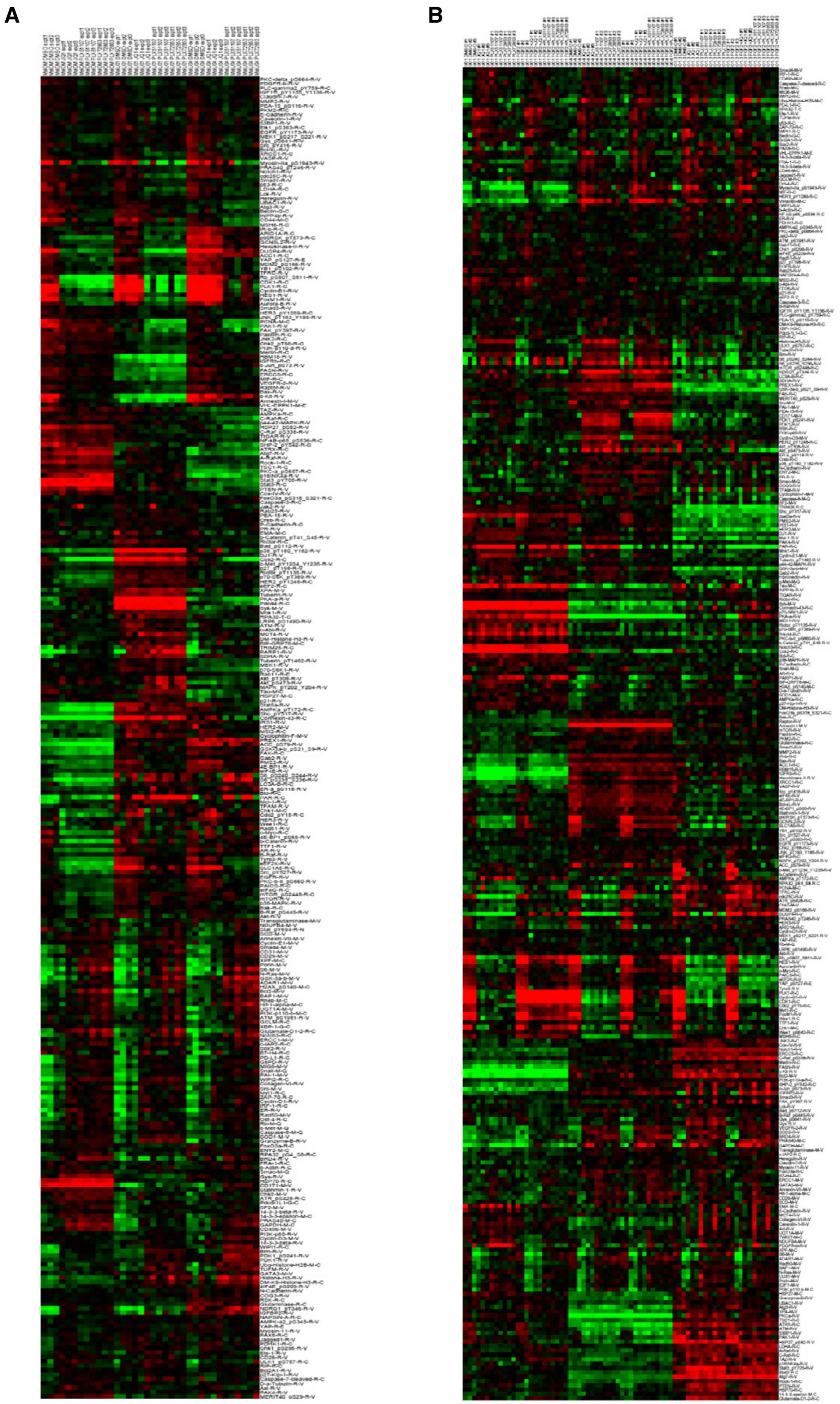

Figure EV1.

© 2019 The Authors

Figure EV2. Effects of BLU9931 on FGF2-mediated resistance to UM cell growth inhibition by BET inhibitors.

UM001 and OMM1.3 cells were treated with 1 μM JQ1, 1 μM PLX51107 or 100 nM PLX72853 in combination with 50 ng/ml FGF2 and 0–10 μM FGFR4 inhibitor BLU9931. UM004 was treated with 2 μM JQ1, 2 μM PLX51107 or 200 nM PLX72853 in combination with 50 ng/ml FGF2 and 0–10 μM BLU9931. Cell growth was determined after 8 days of treatment by crystal violet staining. Fold change in crystal violet stain compared to JQ1 treatment and mean ± SEM of data from triplicate experiments (or $n = 3$) is shown. The unpaired t-test was used. Scale bar: 100 μm.

© 2019 The Authors

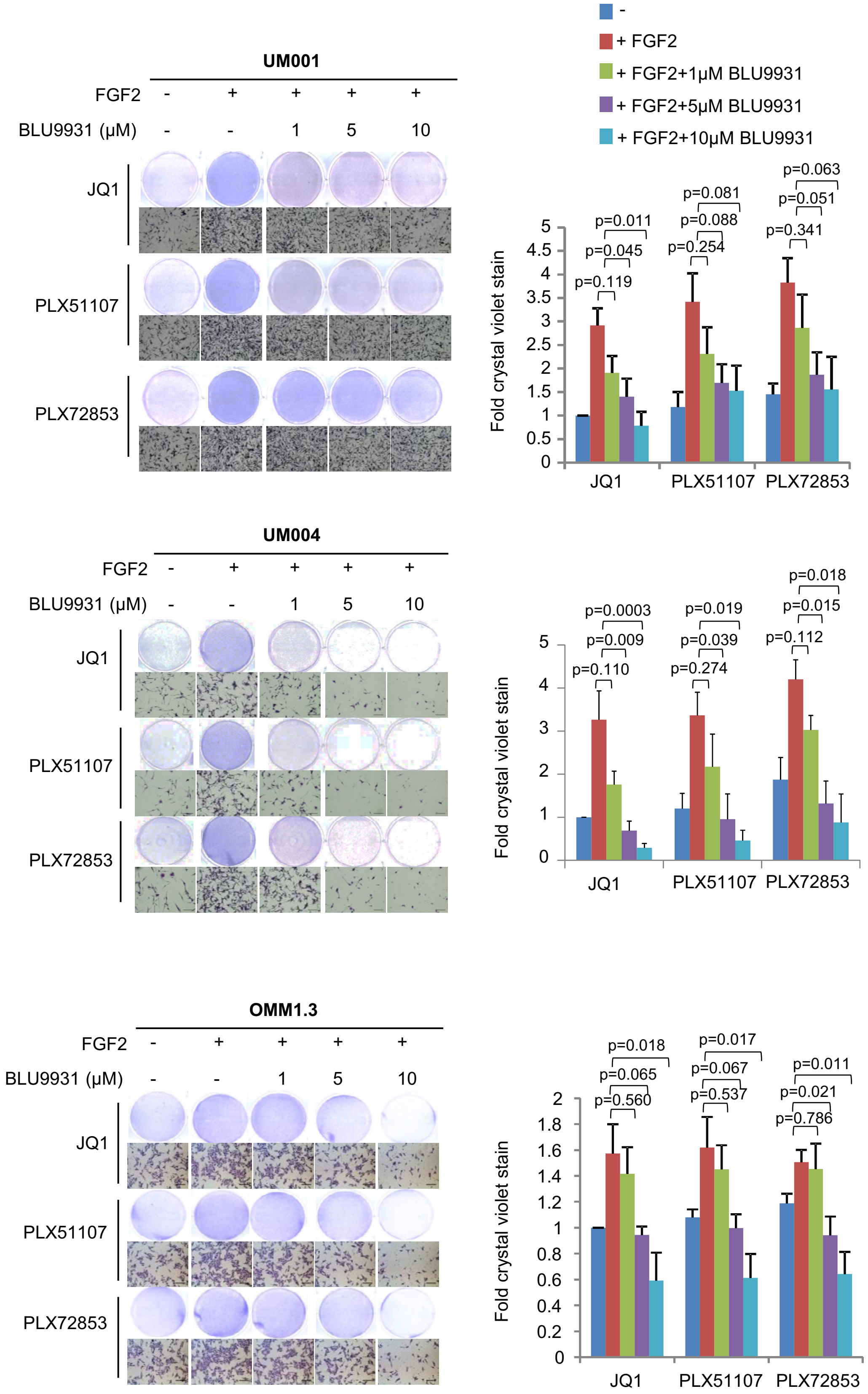

Figure EV2.

 © 2019 The Authors

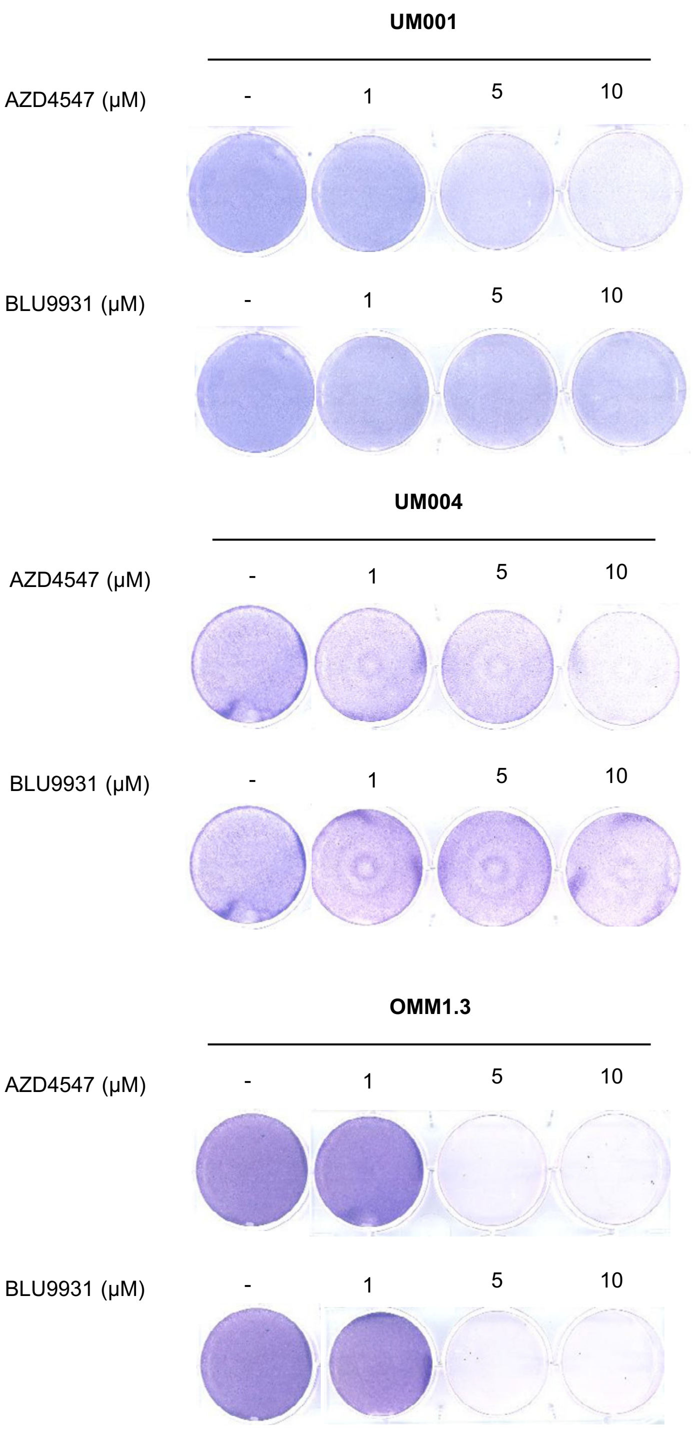

Figure EV3. FGFR inhibitor effects on UM001 colony growth.

AZD4547 and BLU9931 effects on UM cell growth. UM001, UM004 and OMM1.3 were treated with increasing concentrations of AZD4547 or BLU9931. Cell growth was determined after 8 days of treatment by crystal violet staining. AZD4547 and BLU9931 have little/no effect on UM cell growth at 1 μM.

© 2019 The Authors

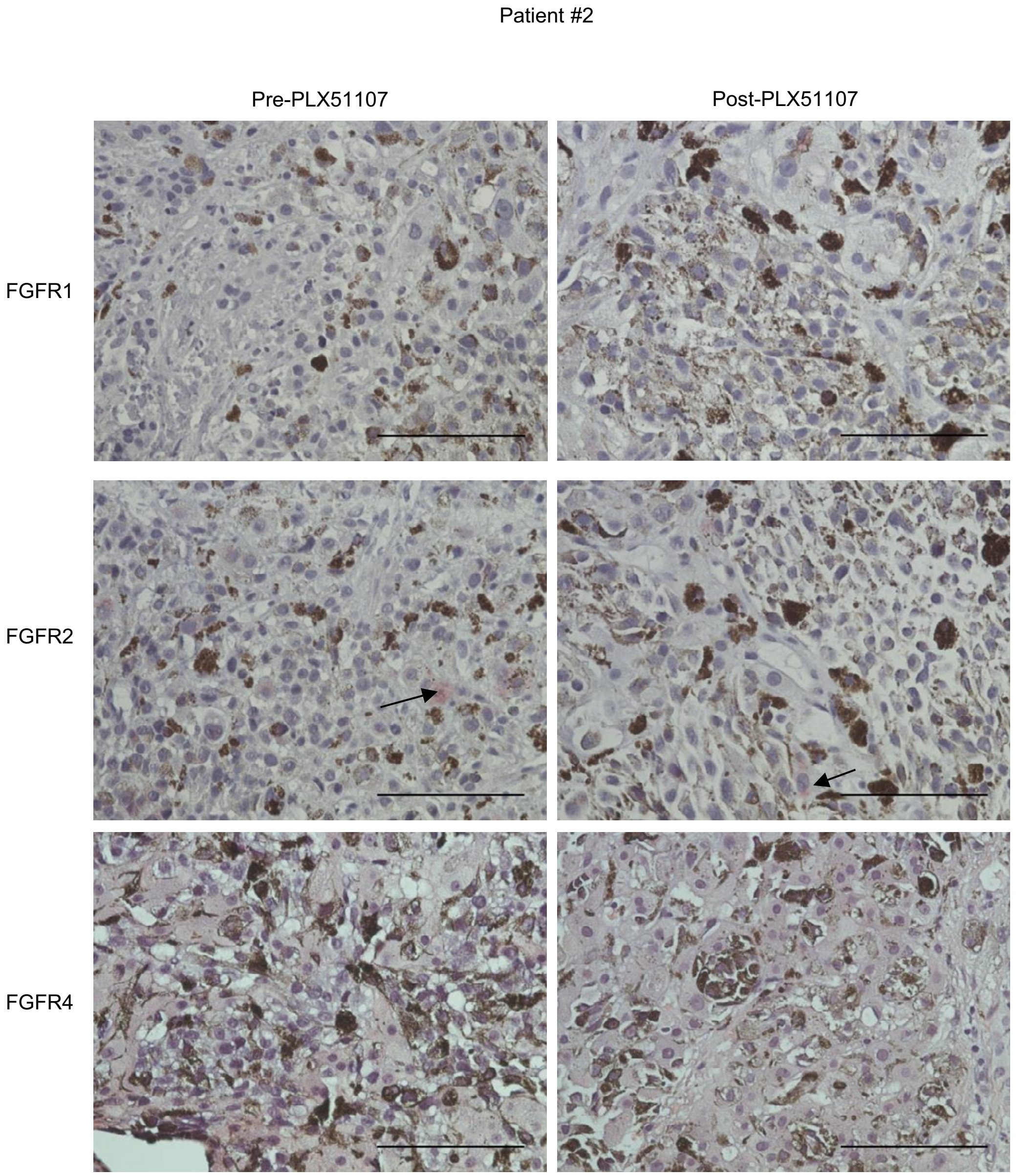

Figure EV4. FGFR staining in patient #2 biopsy (PLX51107 clinical trial).

FGFR1, FGFR2 and FGFR4 staining in pre- and post-PLX51107 treatment tissues isolated from liver metastases of patient #2 enrolled in the PLX51107 clinical trial. Representative images are shown. Arrows are indicating positive FGFR2 staining. FGFR1, FGFR2 and FGFR4 antibody concentrations were 1:25, 1:50 and 1:100, respectively. Scale bar: 100 μm.

 © 2019 The Authors

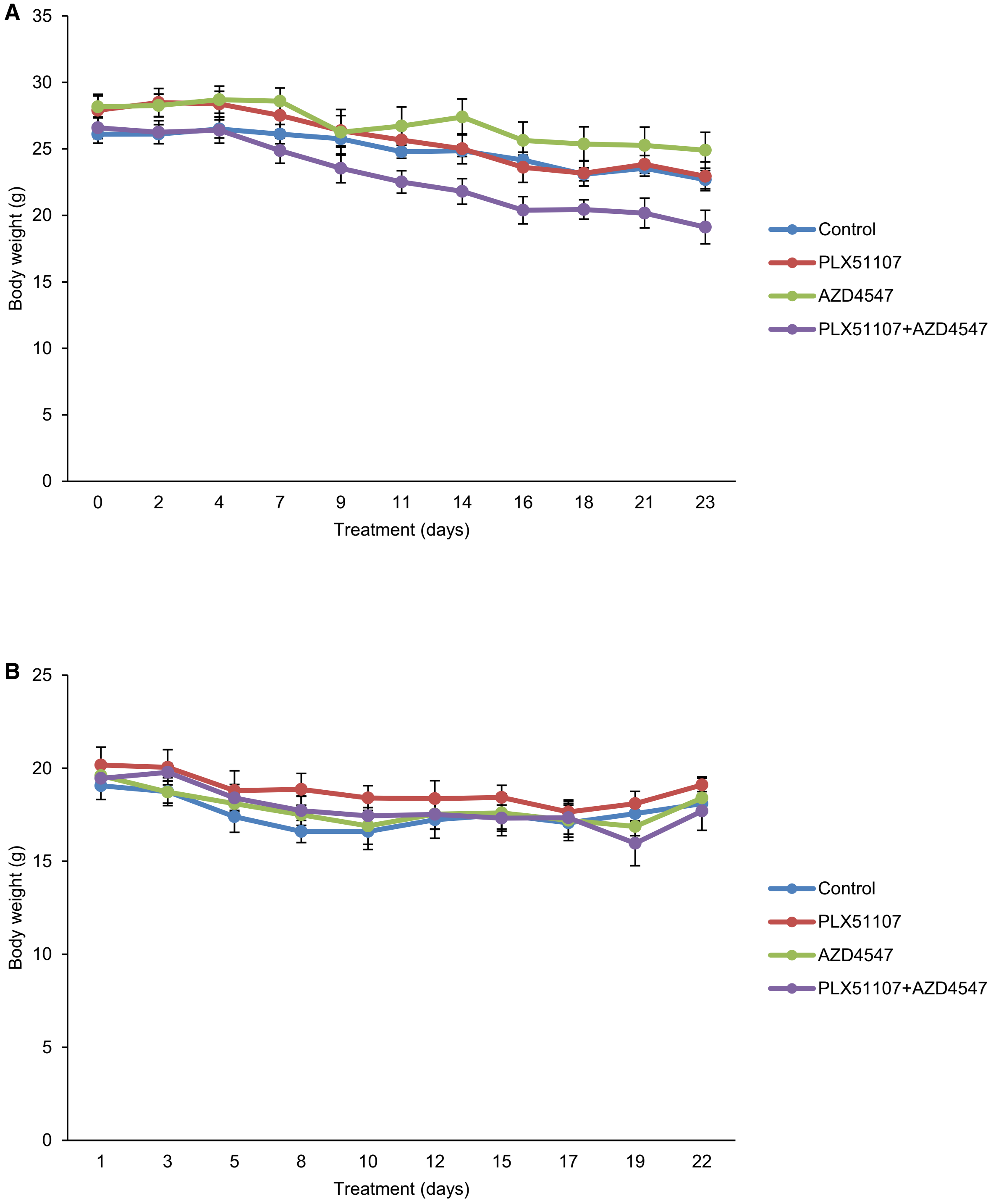

Figure EV5. Weight of animals monitored during the *in vivo* experiments.

A, B (A) Nude mice and (B) NSG mice body weight monitored thrice a week during treatment with 90 mg/kg PLX51107, 5 mg/kg AZD4547, PLX51107 + AZD4547, and DMSO (control).
