# Supplementary material for: Stromal fibroblast growth factor 2 reduces the efficacy of bromodomain inhibitors in uveal melanoma
Source: EMBO Mol Med. 2019 Jan 4;11(2):e9081. doi: 10.15252/emmm.201809081 (PMC6365926; doi:10.15252/emmm.201809081)

**UM001**

Fig 4C

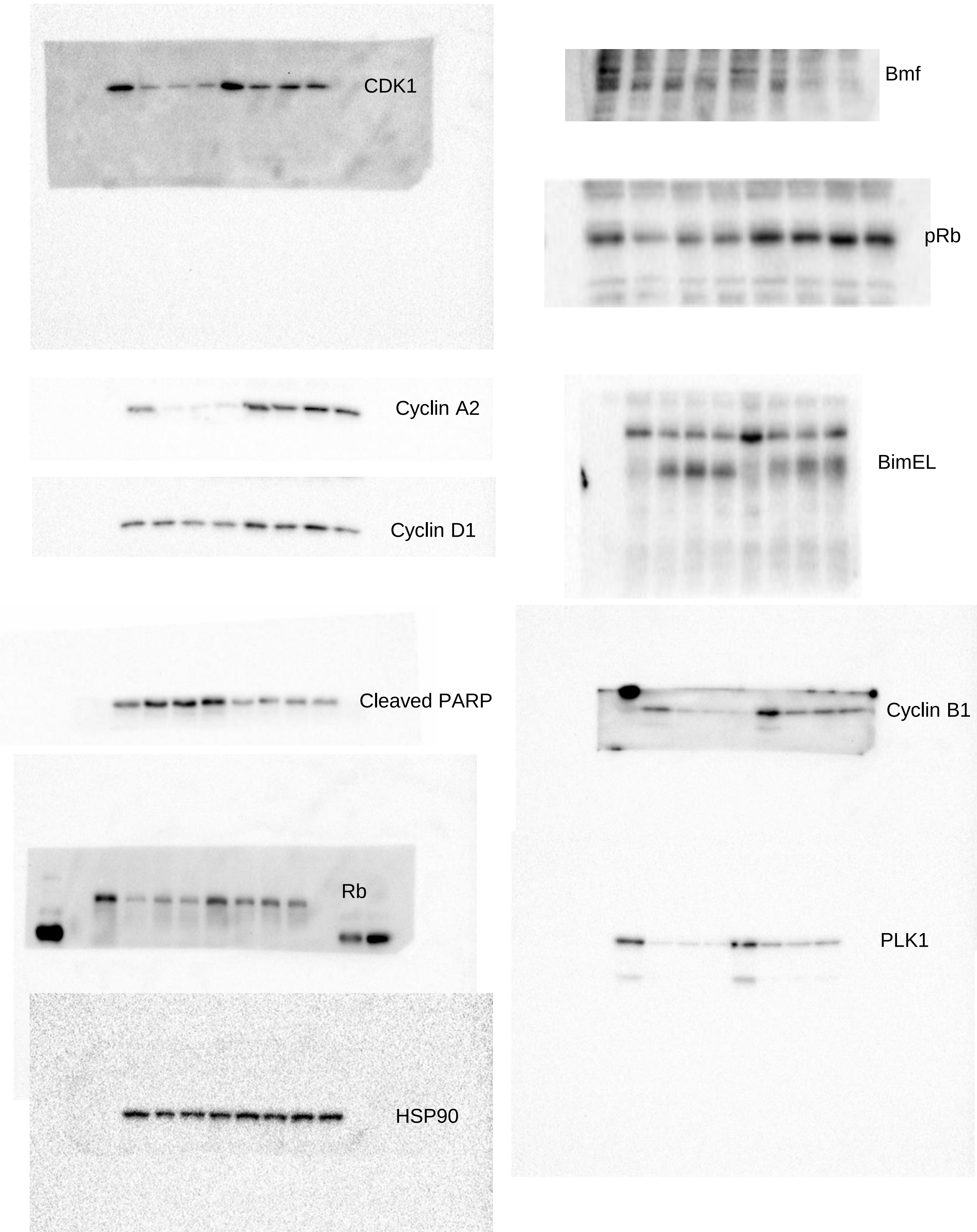
CDK1
Bmf
pRb
Cyclin A2
Cyclin D1
BimEL
Cleaved PARP
Cyclin B1
Rb
PLK1
HSP90

**UM004**

Fig 4C

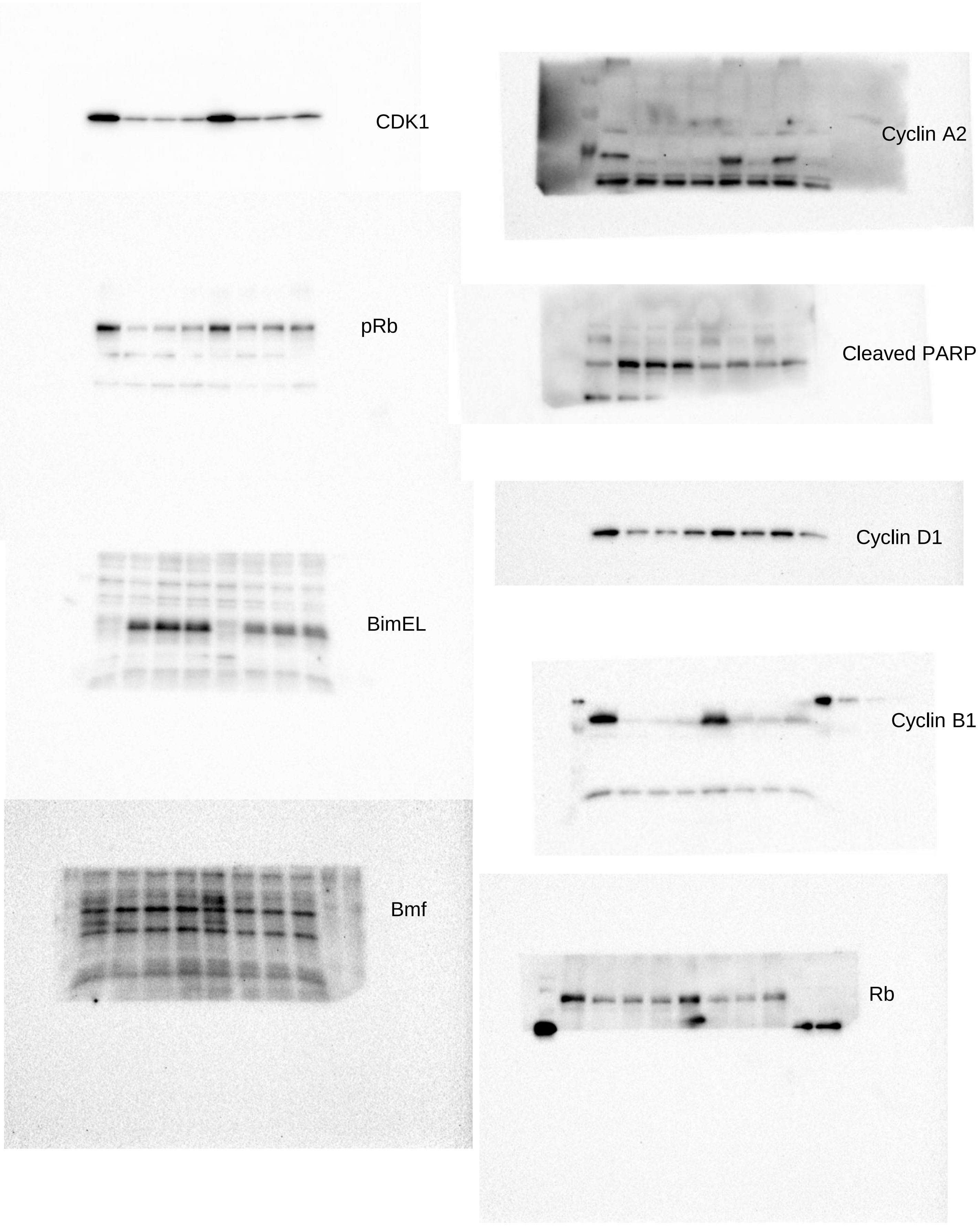
CDK1
Cyclin A2
pRb
Cleaved PARP
Cyclin D1
BimEL
Cyclin B1
Bmf
Rb

**UM004**

Fig 4C

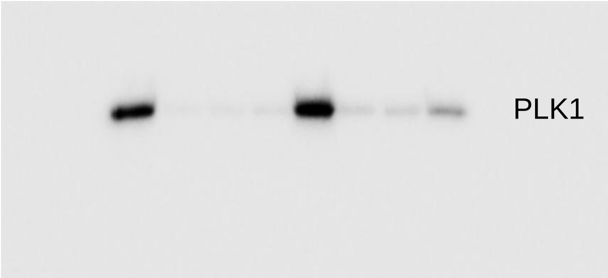


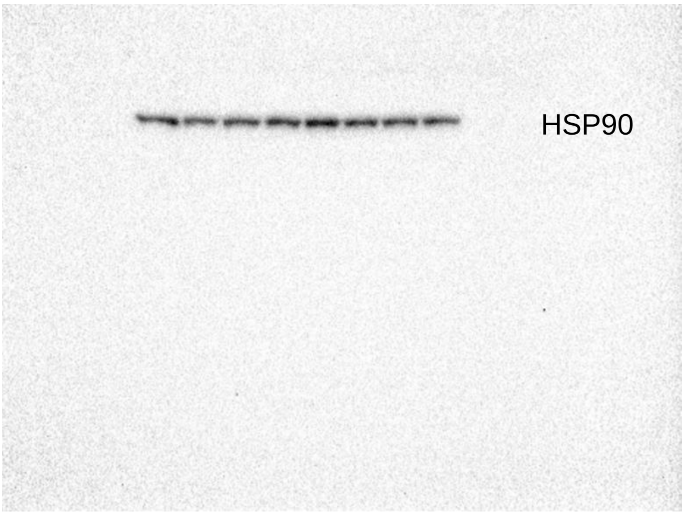

# OMM1.3

Fig 4C

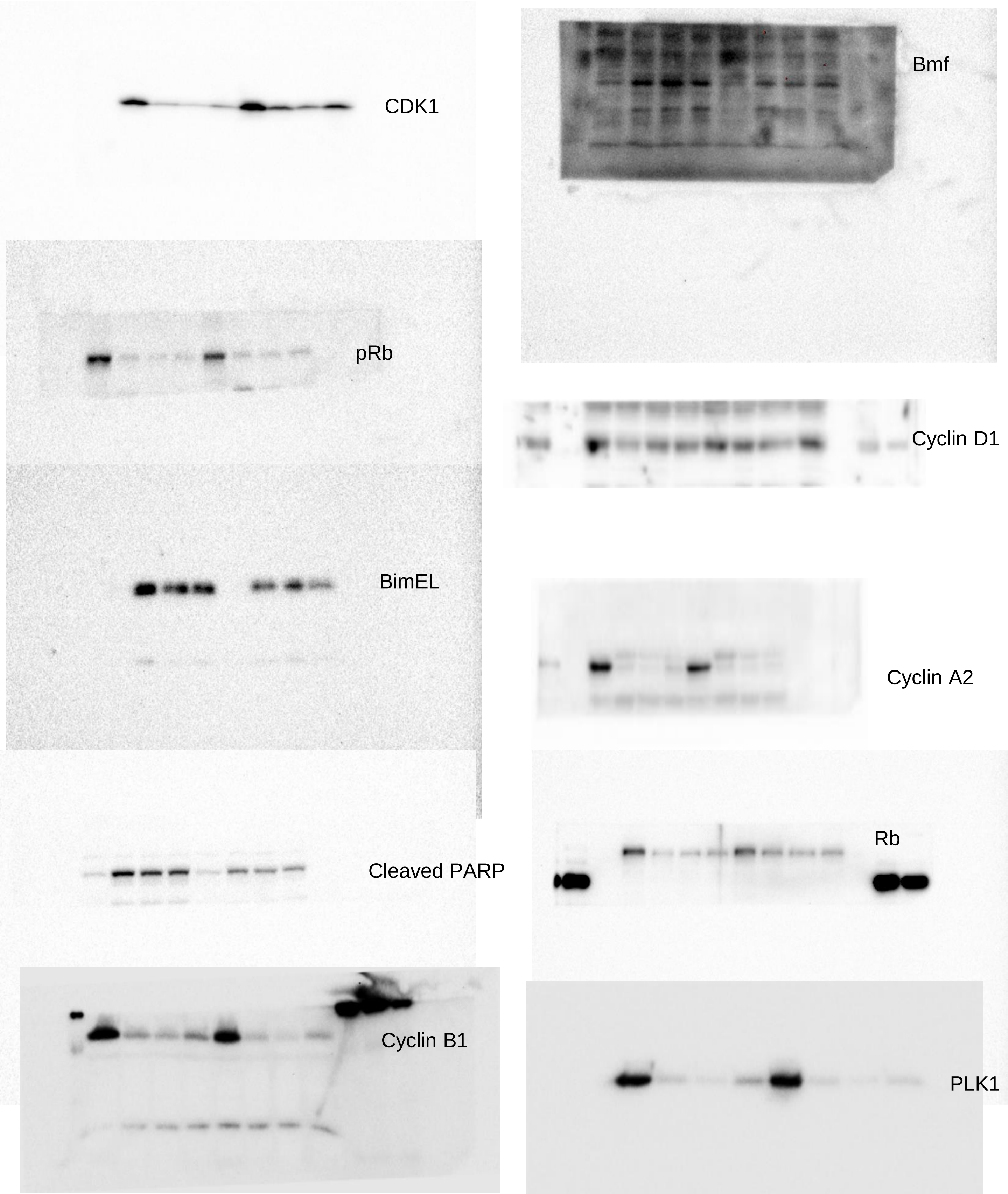
CDK1
Bmf
pRb
Cyclin D1
BimEL
Cyclin A2
Rb
Cleaved PARP
Cyclin B1
PLK1

**OMM1.3**

Fig 4C

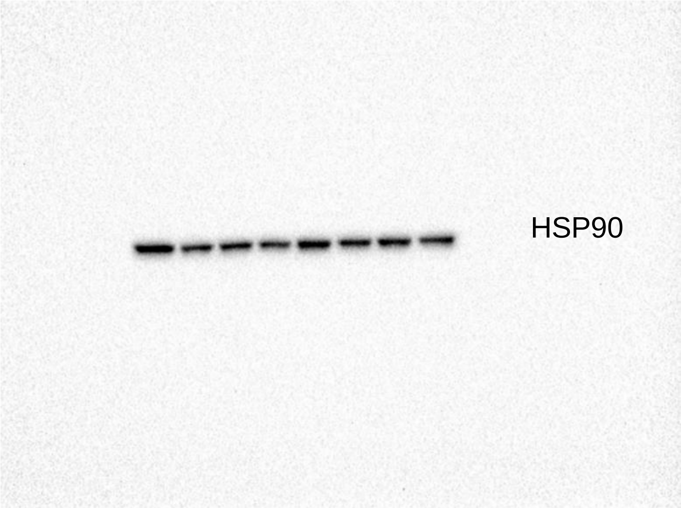

Supplement: Supplementary file 4 — Source Data for Figure 4C [file EMMM-11-e9081-s003.pdf]
