# Supplementary figures and images for: Stromal fibroblast growth factor 2 reduces the efficacy of bromodomain inhibitors in uveal melanoma
Source: EMBO Mol Med. 2019 Jan 4;11(2):e9081. doi: 10.15252/emmm.201809081 (PMC6365926; doi:10.15252/emmm.201809081)

**UM001**

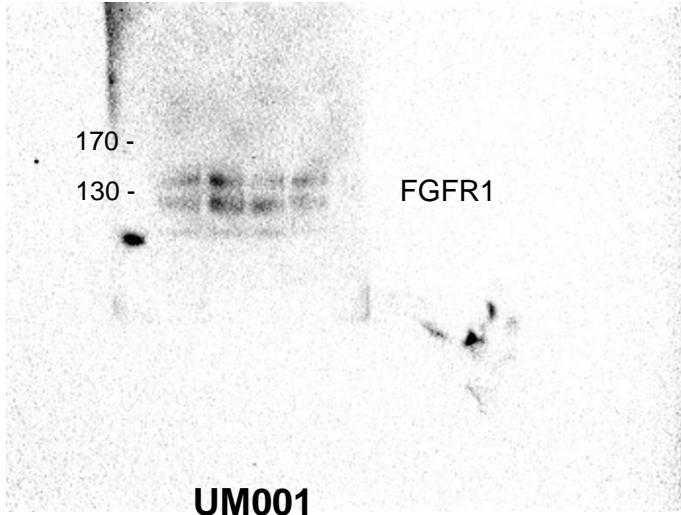

**UM001**

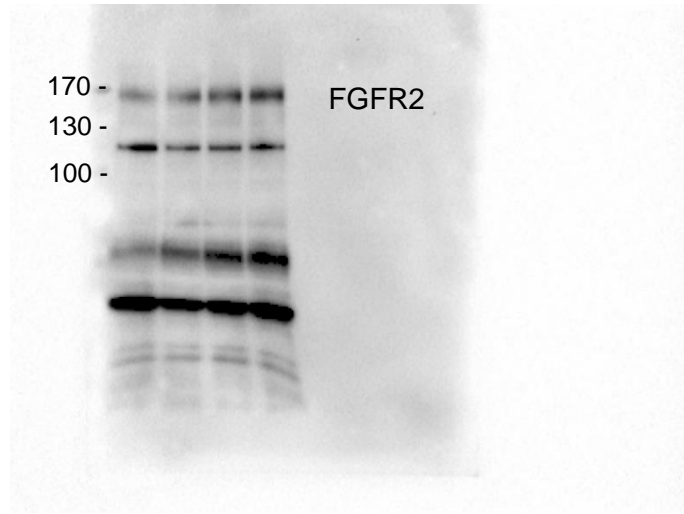

**UM001**

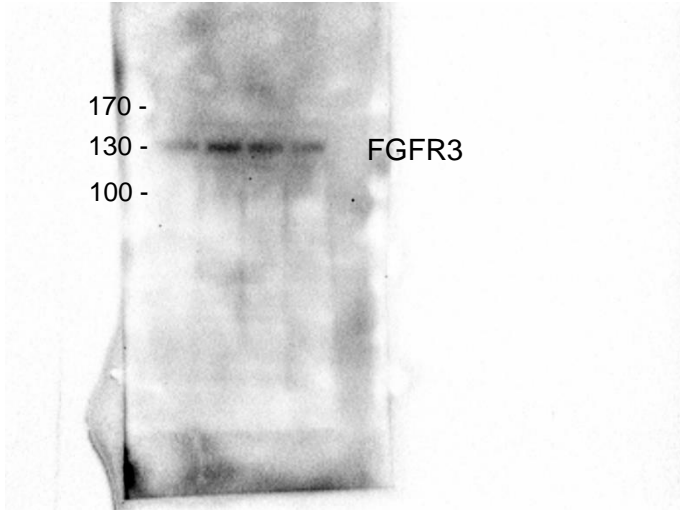

**UM001**

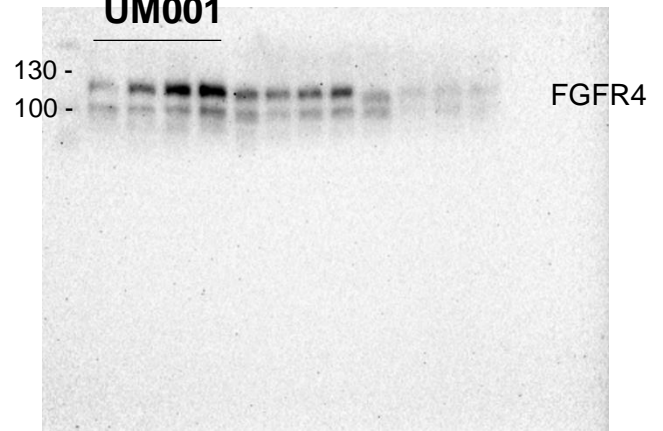

**UM001**

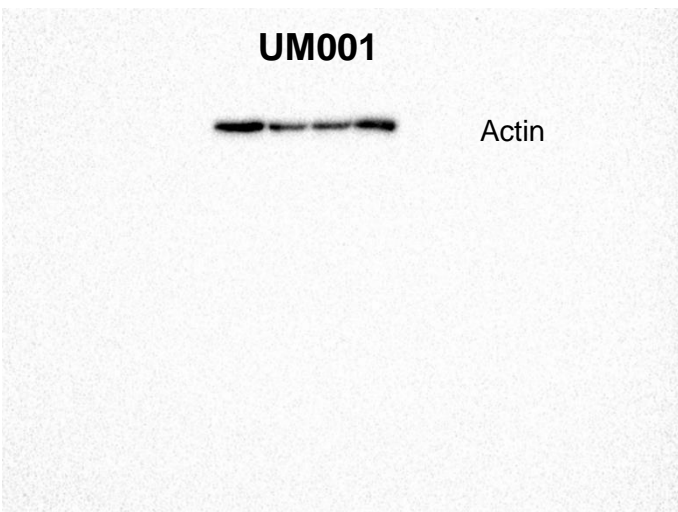

Fig 7B

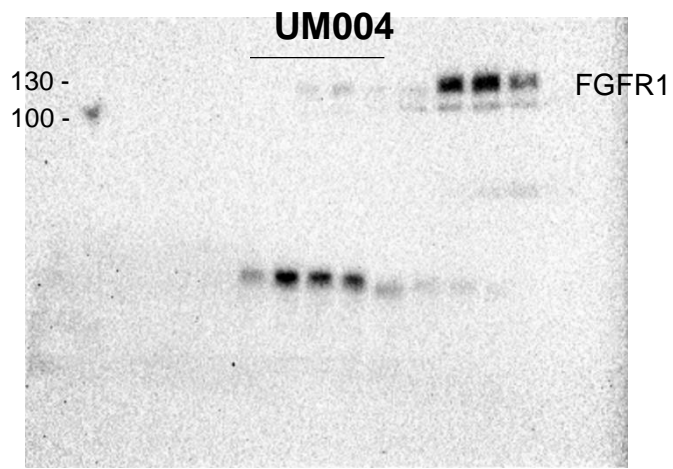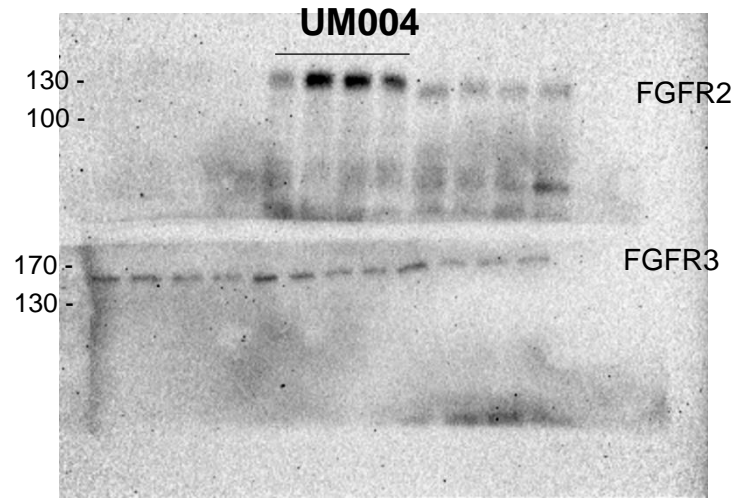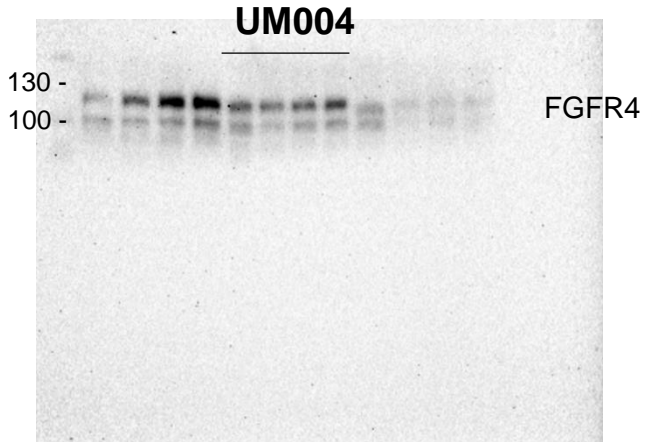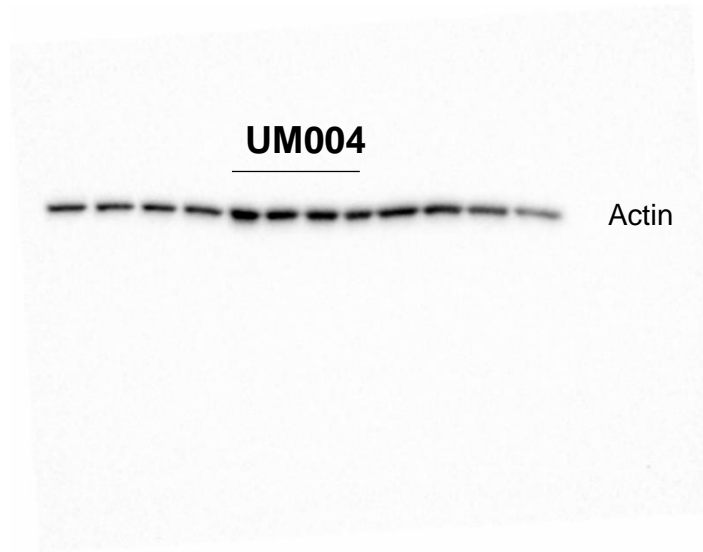

Fig 7B

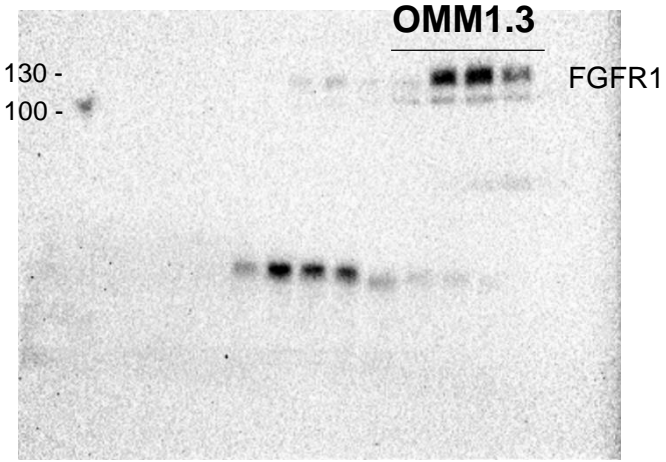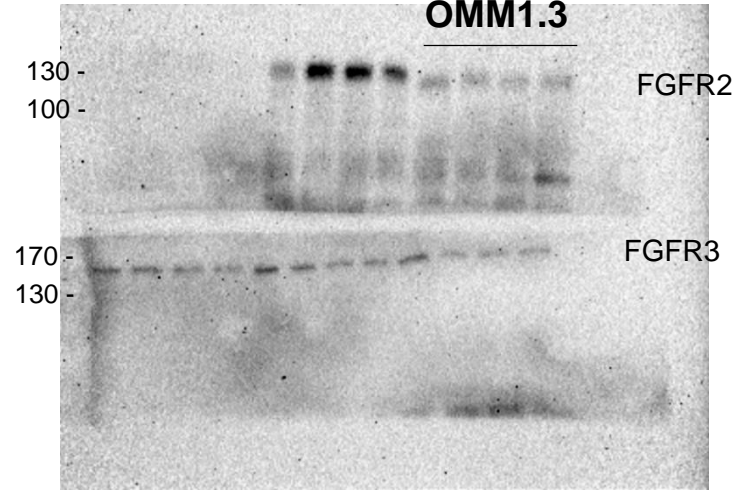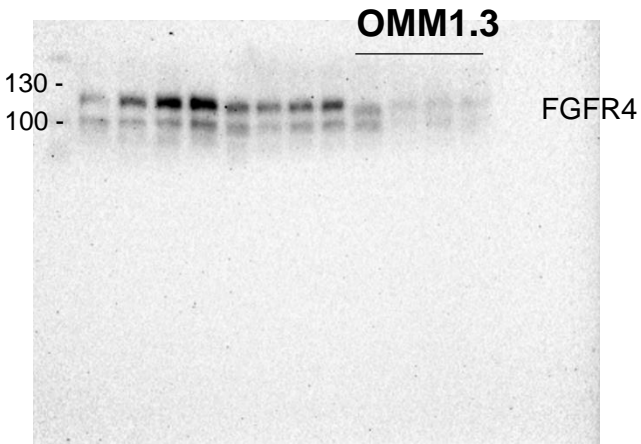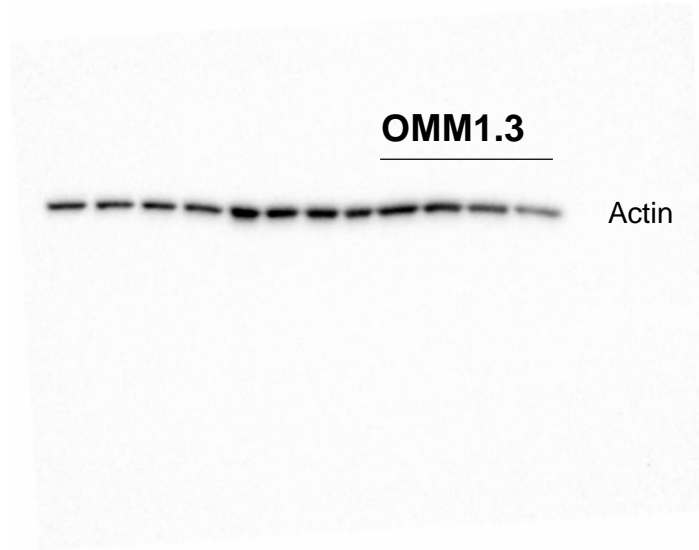

Supplement: Supplementary file 5 — Source Data for Figure 7B [file EMMM-11-e9081-s004.pdf]
